# Donnan effect on chloride ion distribution as a determinant of body fluid composition that allows action potentials to spread via fast sodium channels

**DOI:** 10.1186/1742-4682-8-16

**Published:** 2011-05-30

**Authors:** Sven Kurbel

**Affiliations:** 1 Dept. of Physiology, Osijek Medical Faculty, Osijek, Croatia

## Abstract

Proteins in any solution with a pH value that differs from their isoelectric point exert both an electric Donnan effect (DE) and colloid osmotic pressure. While the former alters the distribution of ions, the latter forces water diffusion. In cells with highly Cl^-^-permeable membranes, the resting potential is more dependent on the cytoplasmic pH value, which alters the Donnan effect of cell proteins, than on the current action of Na/K pumps. Any weak (positive or negative) electric disturbances of their resting potential are quickly corrected by chloride shifts.

In many excitable cells, the spreading of action potentials is mediated through fast, voltage-gated sodium channels. Tissue cells share similar concentrations of cytoplasmic proteins and almost the same exposure to the interstitial fluid (IF) chloride concentration. The consequence is that similar intra- and extra-cellular chloride concentrations make these cells share the same Nernst value for Cl^-^.

Further extrapolation indicates that cells with the same chloride Nernst value and high chloride permeability should have similar resting membrane potentials, more negative than -80 mV. Fast sodium channels require potassium levels >20 times higher inside the cell than around it, while the concentration of Cl^- ^ions needs to be >20 times higher outside the cell.

When osmotic forces, electroneutrality and other ions are all taken into account, the overall osmolarity needs to be near 280 to 300 mosm/L to reach the required resting potential in excitable cells. High plasma protein concentrations keep the IF chloride concentration stable, which is important in keeping the resting membrane potential similar in all chloride-permeable cells. Probable consequences of this concept for neuron excitability, erythrocyte membrane permeability and several features of circulation design are briefly discussed.

## Background

This theoretical paper seeks to interpret similarities in pH, electrolyte and protein compositions of body fluids among diverse animals as requirements imposed by their excitable tissues, particularly neurons and muscle cells.

The logic that follows is based on a previously published argument that similar body fluid osmolarity in various animals is dictated by the opposed Donnan effects of cell proteins and of sodium ions sequestered in the extracellular fluid (ECF) [1]. The conclusion of the cited paper is that the ubiquitous ECF Na^+ ^concentration is determined by the average osmotic burden on animal tissue cells.

## Basic assumptions behind the proposed model of the Donnan effect on body fluid composition

The presence of proteins in any solution exerts two effects on the traffic of ions and water. The first is the electric Donnan effect (DE), which alters the distribution of ions, and the second is the colloid osmotic pressure, which forces water diffusion. Both phenomena are measurable when a protein-rich fluid is in contact with a protein-free fluid through a semipermeable membrane that does not allow protein molecules to diffuse [2].

### Donnan effect (DE)

If protein-bound charges on one side of a semipermeable membrane cannot diffuse through the membrane, the distribution of other ions to which the membrane is permeable is altered [2]. These protein-bound charges are pH dependent, and in the physiological pH range, proteins oveall carry a net negative charge. Thus, they attract cations to enter the protein-rich compartment. Diffusible anions are expelled. The traffic of anions and cations is equally governed by electric fields, concentration gradients and ion-specific membrane permeabilities. Since the accumulation of ions within any cell is followed by osmosis of water molecules, cell edema due to the Donnan effect of cell proteins is prevented through the action of Na^+^/K^+ ^pumps. They expel 3 Na^+ ^and import 2 K^+ ^in every cycle, so some water also leaves the cell. If the membrane permeability for Na^+ ^ions is low, sodium ions are virtually sequestered in the extracellular fluid (ECF), so although sodium ions are not proteins, their sequestered positive charges alter the ion distribution across the cell membrane. This action is analogous to the Donnan effect: ECF sodium ions pull more anions out of the cell. The only way for the cell to reach osmotic equilibrium is to alter its volume until the concentration of nondiffusible intracellular ions (mainly charges on intracellular proteins) is equal to the concentration of ECF-restricted ions (mainly Na^+^) [3].

Owing to the balance of these two opposed Donnan effects, water diffusion is reduced and the osmotic burden on tissue cells is diminished [1].

When the three main body fluid compartments - plasma, interstitial (IF) and cellular fluid - are considered, differences in chloride distribution across cell membranes and capillary walls result not from chloride pumping, but from the Donnan effect of cytoplasmic and plasma proteins [4]. Both cellular and plasma proteins force negative chloride ions to enter the protein-poor IF.

The resting potential of cells that are highly Cl^-^-permeable is more dependent on the cytoplasmic pH value that alters the Donnan effect on chloride ions than on the momentary pumping of sodium and potassium ions. The skeletal muscle resting potential is very close to the chloride Nernst potential since the membrane is highly permeable to chloride ions (10 times higher than to K^+ ^and 1000 times higher than to Na^+ ^[5]). A possible interpretation is that in this setting, any weak (positive or negative) electric disturbances of the resting potential are easily corrected by a chloride shift. If so, the described stabilization of the resting potential is independent of sodium and potassium concentration gradients and this independence of sodium pumping can make it act as an important safety feature against involuntary, or spastic, skeletal muscle contraction.

### Colloid osmotic pressure

The presence of proteins in solution forces water to diffuse from the protein-free compartment and dilute the protein-containing fluid. This pressure is pH-independent and is mainly related to the number of protein molecules acting as particles in solution.

## Description of the proposed model of Donnan effect and body fluid composition

Proteins in any solution with a pH value that differs from their isoelectric point always exert both a DE and colloid osmotic pressure. Since the normal pH of our body fluids ranges from 6.8 (cell fluid) to 7.35 (arterial blood), normal body fluid pH values are much higher than the average amino acid isoelectric point. This makes all proteins in our body fluids negatively charged [2]. So, even when our attention is focused on plasma colloid pressure, or how DE alters ion concentrations in some fluid, we should not neglect the presence of both DE and colloid osmotic pressure in all situations involving proteins in body fluids.

### Expected similar Cl^- ^gradients in tissue cells

The model presented here is focused on excitable cells in which Cl^- ^ions act as stabilizers of their resting membrane potential because of high chloride permeability [2,5]. Various tissue cells probably share similar concentrations of cytoplasmic proteins. They are under almost the same exposure to the IF chloride concentration. So, if the intracellular pH is normal, similar Donnan effects on chloride ions can be expected in all Cl^-^-permeable cells. The consequence is that similar intra- and extra-cellular chloride concentrations make these cells share the same Nernst value for Cl^-^. A further extrapolation is that these cells, with the same chloride Nernst value and high chloride permeability, should have similar resting membrane potentials, more negative than -80 mV.

If cells are surrounded with protein-containing fluid, the Donnan effect of the cytoplasmic proteins is opposed by the same effect of the extracellular proteins. Two opposed Donnan effects result in higher intracellular chloride concentrations, as has been reported in blood cells that normally float in protein-rich plasma [2]. In these cells, sodium pumping is less important for maintaining the cell volume, since the osmotic burden is already reduced by the DE of the plasma proteins. This is a possible interpretation of why the erythrocytes (RBCs) in some carnivores do not have active sodium pumps [6]. A reduced intracellular protein concentration or slightly acidic cytoplasm might allow these cells to maintain normal volume with reduced energy expenditure.

Because of the DE-mediated shift of Cl^- ^ions described above (from protein-rich to protein-poor fluid), the highest chloride concentrations are reported in cerebrospinal fluid (CSF), interstitial tissue fluid and lymph [2,7,8]. Thus, the Donnan effect of plasma proteins enhances capillary filtration and hinders reabsorption of Cl^- ^ions, so interstitial chloride ions are forced to remain in the tissue to be returned to the blood only via lymph drainage, helped by skeletal muscle work. In other words, high plasma protein levels maintain the stability of the IF chloride concentration through their Donnan effect, important in keeping the resting membrane potential similar in all chloride-permeable cells.

**Figure 1:**
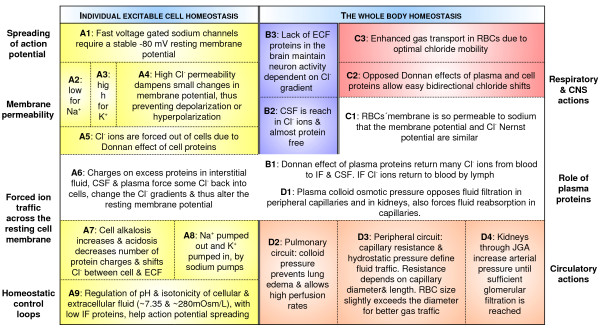
**Schematic display that connects requirements for the fast sodium channel function (field A1) with various aspects of cell physiology (left fields A2 to A9), neuron reactivity (middle top fields B1 to B3), gas traffic in blood (right top fields C1 to C3) or circulation (bottom right fields D1 to D4)**. The pivotal role is reserved for the ECF protein concentrations (central white fields A6, B1, C1, D1) as causes of a local Donnan effect and colloid osmotic pressure. Low IF proteins allow stable resting potentials more negative than -80 mV to be generated. Plasma proteins are optimized to help the chloride shift in RBCs, which is important for gas transport and in pulmonary circulation, peripheral tissue fluid traffic and renal control of arterial pressure.

The model presented here is shown in Fiure 1, which is organized in two sections with four paths. Each path is coded by a different letter and color. The pivotal fields that link cell physiology with body systems have been intentionally left without color code in the center of Figure 1.

### Individual excitable cell homeostasis

The following discussion refers to the left hand side of Figure 1, paths **A1 **to **A9**: from the spreading of action potentials to cellular control of pH and isotonicity in all body fluid compartments and the plasma protein content.

Optimal function of neurons, skeletal and heart muscle cells is a prerequisite for animal mobility and survival. Cellular reactions depend on prompt and well-defined occurrence and spreading of action potentials. Action potential spreading in the aforementioned cell types is mediated through fast, voltage-gated sodium channels. These channels initiate rapid depolarization of the surrounding cell membrane, when the local membrane potential is altered from the resting <-80 to -55 mV or less negative.

Several features are important for the function of fast sodium channels:

Membrane permeabilities to sodium, potassium and chloride ions define the resting membrane potential, as defined by the Goldman equation (Table 1).

**Table 1:** Electric potentials as listed in the Nernst Goldman calculator available at http://nernstgoldman.physiology.arizona.edu/, developed by SH Wright (5)

Ion	Average concentration levels		**Membrane permeability (% of K**^**+ **^**permeability)**	Calculated potentials at normal body temperature (mV)	
			
	intracellular	extracellular		Nernst	Goldman
Skeletal muscle cell (based on 6)					

K^+^	150	4.5	100	-93.7	-88.3
	
Na^+^	12	145	1	+66.6	
	
Cl^-^	4.2	116	1000	-88.6	

Red blood cell (based on 7)					

K^+^	140	4.5	100	-91.8	-14.3
	
Na^+^	11	145	54	+68.9	
	
Cl^-^	80	116	21	-9.9	

A sufficiently low resting potential that allows the sodium channels to function (Figure 1, **A1**) can be achieved only if the membranes are almost impermeable to Na^+ ^(field **A2**), since any substantial sodium current would make the resting membrane potential less negative than -80 mV, and this would leave sodium channels inactive after repolarization.

Combined high permeability for potassium (field **A3**) and chloride (field **A4**) moves the resting membrane potential to the required range, more negative than -80 mV, if the concentration gradients for these two ions are sufficient:

- K^+ ^concentrations: low outside and high inside the cell, maintained by Na/K pump function;

- Cl^- ^concentrations: high outside and low inside the cell, due to the Donnan effect of cytoplasmic proteins (fields **A4 **and **A5**), which requires normal cytoplasmic pH.

Sufficient potassium gradients are associated with a K^+ ^Nernst potential more negative than -80 mV. This means that the K^+ ^concentration needs to be >20 times higher inside the cell than outside. The situation is similar to that of Cl^- ^ions: >20 times higher concentration is required outside the cell to make the Nernst potential for chloride more negative than -80 mV. This means that a high concentration of chloride ions is needed in the IF and of potassium ions in the cytoplasm. Without these gradients, the resting membrane potential would be less negative than -80mV and this would compromise the spreading of action potentials through the activation of fast sodium channels. When osmotic forces, electroneutrality and other ions are taken into account with >20 times ionic gradients, the overall osmolarity needs to be near 280 to 300 mosm/L, or isotonic (field **A9**). In short, the necessity for a stable resting potential more negative than -80 mV in our excitable tissues determines body fluid osmolarity.

The Donnan effect of cytoplasmic proteins depends on the intracellular pH. The electric fields surrounding cell proteins are less strong if there is intracellular acidosis. This diminishes the effect of proteins on the ion distribution within the cell and in its vicinity (field **A7**). A similar action is exerted if the extracellular protein concentration is excessive (field **A6**). A possible extrapolation is that the requirement for a stable resting potential in our excitable tissues might have determined the low concentration of IF proteins, making animal capillaries less protein-permeable. The same argument stands for the fine control of cellular and extracellular pH values (field **A9**).

### Whole body homeostasis

The following discussion refers to the right hand side of Figure 1, paths **B1 **to **B3**: the roles of the protein-poor cerebrospinal fluid in maintaining neuronal excitability.

The high permeability of neurons to chloride ions [5] positions their resting potential close to the Nernst potential of chloride ions. A high chloride gradient across their membranes is determined by the DE of the cytoplasmic proteins; there are almost no extracellular proteins in the surrounding IF (field **B2**). This setting allows higher Cl^- ^concentrations to be established in the cerebrospinal fluid and interstitial fluid than in the plasma. The consequence is that similar chloride gradients are expected in all neurons with normal cytoplasmic pH, making their resting potentials similar and stable (field **B3**). This stability can be compromised under the following conditions:

Diseases that accumulate extracellular proteins behind the blood brain barrier can alter neuron function through an altered gradient of Cl^- ^ions across their membranes (reduced IF chloride and increased cellular chloride concentrations).

In edematous neurons, more cellular water dilutes the cell proteins, so the charges are less dense, and this allows more chloride to remain inside. This makes the resting membrane potential less negative.

The following discussion relates to the right hand side of Figure 1, paths **C1 **to **C3**: the transport of gases, the chloride shift, and the membrane permeability of red blood cells.

The path describes the effect of RBC membrane permeability on the chloride shift and transport of gases:

Gas transport in RBCs is helped by the chloride shift through the erythrocyte membrane every time an RBC enters pulmonary or peripheral capillaries [2]. Since there is two-way traffic of chloride ions, Cl^- ^ions enter or leave erythrocytes without difficulty; evolution developed RBCs with membranes permeable to sodium (field **C1**). The consequence is that the normal RBC membrane potential is close to the Nernst potential of these cells for chloride ions [7]. In this way, even small electric fields can be compensated through Cl^- ^traffic and this enhances the capacity of RBCs for gas transport (fields **C2 **and **C3**).

The following discussion relates to the right hand side of Figure 1, paths **D1 **to **D3**: plasma colloid osmotic pressure, capillary permeability and the design of the circulation.

This path describes the effect of plasma colloid osmotic pressure on circulatory design:

In some fish, the capillaries are so permeable to proteins [9] that the IF protein content is similar to that of the plasma. A possible speculation is that with no Donnan effect to alter the IF ion composition, and no colloid osmotic pressure to oppose plasma filtration, a much lower hydrostatic pressure can be sufficient for capillary wall filtration. Capillaries under low hydrostatic pressure need to be wide to provide sufficient perfusion. This means that RBCs can and should be large, as found in poikilothermous animals that also share low systemic circulatory pressures [10]. In these low-pressure animals, most of the filtered fluid is recirculated to the blood via the lymphatics, since there is no large colloid pressure gradient between the plasma and IF. Lymph recirculation is often helped by slow-action lymphatic hearts.

The reported higher arterial pressures in terrestrial amphibians [11] can be attributed to the greater perfusion needs of their skeletal muscles. Probably, the danger of a detrimental surplus of filtered tissue fluid, due to increased perfusion pressures, led to the development of animals with less permeable capillaries. The next probable step was the introduction of almost protein-impermeable capillaries, which allowed circulatory pressures and perfusion rates to be much higher, since the plasma colloid osmotic pressure opposed filtration and thus prevented edema. Further refinements came with reduced RBC size, which allowed the capillaries to be narrow and more resistant. The drop in hydrostatic pressure along capillary beds allowed interstitial fluid to be reabsorbed through capillary segments in which the hydrostatic pressure was below the plasma colloid osmotic pressure (field **D1**). The consequence for mammals is that lymphatics are less dense and carry only some 10% of the tissue fluid back to the circulation, while the rest is reabsorbed by the peripheral capillaries.

Although it might seem that higher concentrations of plasma proteins mean that even greater hydrostatic pressure and higher perfusion rates can be applied in narrow capillaries without risking tissue edema, a ceiling on plasma protein concentration is imposed by the kidneys (field **D4**). More proteins require even higher arterial pressures to ensure the expected 20% filtration fraction in the kidneys. The compromise plasma protein content, shared by humans and various other animals, is near 70 g/L, combined with a mean arterial pressure near 90 mmHg. This setting seems to provide skeletal muscle perfusion pressures that should suffice to survive the expected challenges before procreation.

This optimal level of plasma proteins is matched by an adequately low capillary wall permeability for proteins, allowing the perfusion rates in pulmonary and peripheral capillaries to be high without developing pulmonary or peripheral tissue edema (fields **D2 **and **D3**).

### Extrapolations of the proposed model

The model presented here is focused on interactions that link seemingly unrelated aspects of our circulation in concordance with the reported densities of sodium pumps: a small number on red blood cells and a high density on neurons and muscle cells [12]. The different relative densities of sodium channels allow some predictions to be made:

Most excitable cells depend on sodium channels for action potential spreading so their resting potentials are anchored by the Cl^- ^Nernst potential value, which allows chloride shifts to occur easily in both directions. This feature of cell membranes reduces the chances of accidental action potential propagation. A possible important exception might be the heart pacemaker cells. Their unstable resting membrane potential depends on high sodium permeability [13,14] and it is less negative than required for functional voltage-gated sodium channels, so the occurrence and spreading of action potentials rely on calcium channels and the Na^+^/Ca^++ ^exchanger. For pacemaker cell function, Cl^- ^permeability seems less important than Na^+^/K^+ ^pumping, making these cells more sensitive to ouabain exposure than to internal acidosis.

If the plasma protein level allows higher pressures and perfusion rates to develop in skeletal muscle capillary beds, a relationship is expected between hemoglobin and albumin levels in different animals. Normal blood value data can be found for mammals [15], but unfortunately, no similar list of normal blood values for amphibians and reptiles seems to be available.

**Figure 2:**
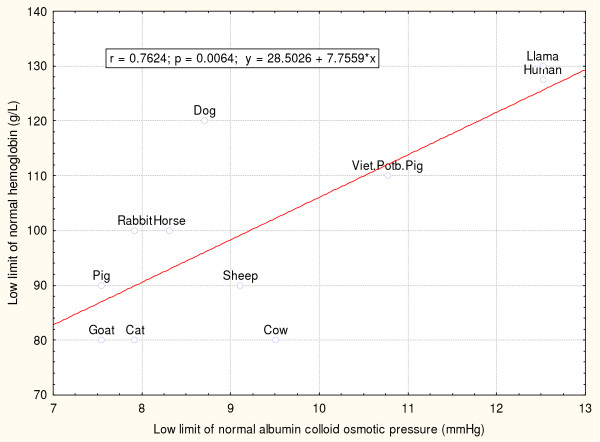
**Relation between albumin-induced colloid osmotic pressure and normal hemoglobin values in human and mammals (based on data from **[15]**and formula from **[16]). The linear correlation suggests that animals with higher hemoglobin values in the blood also tend to have higher albumin-induced plasma colloid pressure values.

Figure 2 shows a simple linear correlation between the low limits of normal albumin and hemoglobin values in 10 animals and humans (albumin data from [15] were used to calculate the colloid pressure according to the formula from [16]). Higher blood hemoglobin levels are associated with higher albumin levels, showing that increased blood capacity for oxygen is associated with higher plasma colloid osmotic pressure. Higher hemoglobin values allow faster transit of RBCs through the capillaries, while higher colloid osmotic pressure prevents excessive fluid filtration through the capillary walls. This correlation suggests that regulatory loops of erythropoiesis and protein metabolism optimize the deliveries of fluid and gases in peripheral tissues.

## References

[B1] Kurbel S Are extracellular osmolality and sodium concentration determined by Donnan effects of intracellular protein charges and of pumped sodium? J Theor Biol 2008 252 769 72 10.1016/j.jtbi.2008.02.022 18374361

[B2] Ganong WF Review of Medical Physiology 2005 22 New York: McGraw-Hill Medical 51 82 580, 593, 612-4, 670

[B3] Schultz SG Johnson LR The internal environment Essential Medical Physiology 2003 3 San Diego: Academic Press 5 6

[B4] Baumgarten CM Feher JI Sperelakis N Osmosis and regulation of cell volume Cell Physiology Sourcebook: A Molecular Approach 2001 3 San Diego: Academic Press 339

[B5] Wright SH Generation of resting membrane potential Adv Physiol Educ 2004 28 139 142 10.1152/advan.00029.2004 15545342

[B6] Sarkadi B Parker JC Activation of ion transport pathways by changes in cell volume Biochim Biophys Acta 1991 1071 407 427 1721542 10.1016/0304-4157(91)90005-h

[B7] London RD Lipkowitz MS Sinert RH Abramson RG Modulation of ionic permeability in a nonpolarized cell: effect of cAMP Am J Physiol 1989 257 6 Pt 2 F985 93 2557768 10.1152/ajprenal.1989.257.6.F985

[B8] Migliore A Paoletti P Villani R Studies on the passage of water, electrolytes and proteins into the cerebrospinal fluid in the human Acta Neurochir (Wien) 1964 12 1 10 10.1007/BF01404698 14207523

[B9] Hargens AR Millard RW Johansen K High capillary permeability in fishes Comp Biochem Physiol A Comp Physiol 1974 48 675 80 10.1016/0300-9629(74)90610-0 4152024

[B10] Snyder GK Sheafor BA Red blood cells: centerpiece in the evolution of the vertebrate circulatory system Amer Zool 1999 39 189 198

[B11] Hicks JW The physiological and evolutionary significance of cardiovascular shunting patterns in reptiles News Physiol Sci 2002 17 241 5 12433978 10.1152/nips.01397.2002

[B12] Clausen T Clinical and therapeutic significance of the Na+,K+ pump Clin Sci (Lond) 1998 95 3 17 10.1042/CS19970254 9662481

[B13] Seyama I Bonke FIM Which ions are important for the maintenance of the resting membrane potential of the cells of the sinoatrial node of the rabbit? The Sinus Node 1978 The Hague: Nijhoff 339 347

[B14] Kurbel S Simplified interpretation of the pacemaker potential as a tool for teaching membrane potentials Adv Physiol Educ 2003 27 159 61 10.1152/advan.00054.2002 12928327

[B15] Duncan JR Prasse KW Veterinary Laboratory Medicine 1986 2 Iowa State University Press

[B16] Landis EM Pappenheimer JR Exchange of substances through the capillary walls Handbook of Physiology Vol. 2, Sec. 2 1963 Washington, DC: American Physiological Society 961 1034

